# LIFE COURSE “COGNABILITY”: INVESTIGATING NEIGHBORHOODS AND COGNITIVE HEALTH RISKS ACROSS THE LIFESPAN

**DOI:** 10.1093/geroni/igae098.1500

**Published:** 2024-12-31

**Authors:** Jessica Finlay, Grace Savard, Desiree Alvarez-McNelis, Mallory Sagehorn, Michael Esposito

**Affiliations:** University of Colorado Boulder, Boulder, Colorado, United States; University of Minnesota; University of Minnesota, Minneapolis, Minnesota, United States; University of Colorado Boulder, Boulder, Colorado, United States; University of Minnesota, Minneapolis, Minnesota, United States

## Abstract

A growing body of evidence identifies potentially modifiable individual risk factors for Alzheimer’s Disease and Related Dementias (ADRD) such as physical inactivity, hypertension, and social isolation. However, the neighborhood built and social environments in which people develop and navigate these risk factors are often overlooked. This knowledge gap is particularly acute for longitudinal neighborhood contexts that may differentially structure opportunities and barriers to individual ADRD risk across the lifespan. The Neighborhoods and Health at All Ages Study employed seated and mobile interviews from August 2023-March 2024 to investigate which neighborhood amenities and hazards are perceived as most relevant to cognitive health-promoting activities at different life course stages. Fifty-six study participants living across the Minneapolis-St. Paul (MN) metropolitan area were on average 42 years old (range: 18-75). 52% identified as female, 40% male, 8% non-binary; and 29% identified as Asian, 24% Hispanic, 19% Black/African American, 17% non-Hispanic White, and 11% American Indian/Alaska Native. Reflexive thematic analysis identified that greater access to affordable amenities, online social infrastructure, and robust public and active transit in young adulthood; safe transportation, online neighborhood swaps, and educational sites (e.g., daycares, schools) in mid-life; and civic/social organizations, arts/cultural amenities, and accessible outdoor recreation in later life can support cognitively healthy lifestyle behaviors. The interviews captured nuanced perspectives and varying personal experiences of how individuals uniquely engage with their neighborhood environments, including navigating structural racism, classism, and ageism. Results may inform upstream community-level interventions to help create more equitable neighborhoods with opportunities to support lifelong cognitive health.

